# Cancer drug pan-resistance: pumps, cancer stem cells, quiescence, epithelial to mesenchymal transition, blocked cell death pathways, persisters or what?

**DOI:** 10.1098/rsob.120066

**Published:** 2012-05

**Authors:** Piet Borst

**Affiliations:** Molecular Oncology, NKI-AVL, Plesmanlaan 121, Amsterdam, The Netherlands

**Keywords:** drug resistance, multi-drug resistance, residual disease, persisters, epithelial to mesenchymal transition, pan-resistance

## Abstract

Although chemotherapy of tumours has scored successes, drug resistance remains the major cause of death of cancer patients. Initial treatment often leaves residual disease, from which the tumour regrows. Eventually, most tumours become resistant to all available chemotherapy. I call this pan-resistance to distinguish it from multi-drug resistance, usually describing resistance caused by upregulation of drug transporters, such as P-glycoprotein. In this review, I discuss mechanisms proposed to explain both residual disease and pan-resistance. Although plausible explanations are at hand for residual disease, pan-resistance is still a mystery. My conclusion is that it is time for a major effort to solve this mystery using the new genetically modified mouse tumour models that produce real tumours resembling cancer in human patients.

## 2. Introduction

Most patients with disseminated cancer die because their metastases become resistant to all available drugs. Often resistance arises in two steps. Initially, the tumour responds to the drug, but not all tumour cells are killed. This residual disease eventually gives rise to tumours that do not respond to any drug anymore [1]. I call this pan-resistance, a term borrowed from bacteriology [2], to distinguish it from multi-drug resistance (MDR), classically used to describe resistance caused by promiscuous drug transporters. These can extrude many drugs from cancer cells, but not all, and they can therefore not cause pan-resistance. As we learn more about cancer and about mechanisms of resistance, explanations for residual disease and for pan-resistance of tumours in the terminally ill have proliferated. Here I discuss the evidence for these explanations. Although residual disease and pan-resistance are fundamentally different, the mechanisms causing resistance overlap. I therefore use residual disease as the overture to the main topic, pan-resistance.

Drug resistance is a vast research field. To stay within the word count allotted, my treatment of the subject is therefore selective, if not idiosyncratic. To economize on references, I liberally quote reviews that I consider sound.

## 3. Some introductory comments on drug resistance

Often investigators think about drug resistance of cancer cells in the same terms as drug resistance of bacteria. Well-behaving bacteria should be sensitive to the antibiotics that we aim at them. They may become resistant by mutation or adaptation; they may hide in body sanctuaries, where the drugs do not reach them; but basically, if we use the right drug, the bacterium should respond and die. To transpose this expectation to cancer cells without modification is unrealistic. Cancer cells are body cells that misbehave; basically, their proteins and metabolic pathways are the same as in normal host cells. With few exceptions, cancer cells do not have enzymic reactions that are completely absent in normal cells, like cell wall synthesis, which can be targeted in bacteria without hitting mammalian cells. The ground state of cancer cells is resistance, not sensitivity. If drugs hit cancer cells more than do normal cells, it is only because the cancerous state has entailed cellular changes that make the cell more vulnerable. The term ‘primary drug resistance’ for tumours that do not respond at all is therefore misleading. The cells are not resistant, they are just not more sensitive to the drug than normal cells. Tumour cells that do respond are more vulnerable than normal cells and are really hypersensitive to the drug, as first pointed out by the British medical oncologist Adrian Harris [3]. If hit by a drug in that Achilles heel [4,5], the tumour cell may develop secondary resistance, levelling the playing field and reducing its drug sensitivity to that of normal cells. The general belief in the cancer field is that tumours have a sufficient number of Achilles heels that we shall eventually be able to destroy any tumour anywhere in the body. Maybe; let us hope so.

Obviously, drugs can work only when they reach the tumour cells. That does not always occur. There are sanctuaries in the body, where tumour cells are hard to reach. An interesting recent example is provided by the results of neo-adjuvant therapy of breast cancer, used to shrink the tumour before operation. The tumour may have disappeared from the breast by this treatment, but in some cases, metastases appear later in the brain [6] (S. Rodenhuis 2012, personal communication), where tumour cells have escaped from the drugs given to the patient by hiding behind the blood–brain barrier [7]. There are also well-documented cases of tumours that are poorly penetrable by drugs, because of a massive stromal component (e.g. pancreatic cancer [8,9]) or elevated intratumoral pressure. These ‘mechanical forms’ of resistance will be disregarded here. This review deals with biochemical mechanisms of resistance. This does not imply that tumour cells are impervious to their surroundings. However, the evidence that the interaction with stromal cells affects the resistance of tumour cells is not compelling, in my opinion. Notably, the notion that chemotherapeutic agents may act by inducing an immune response against the tumour [10] is not supported by evidence from realistic non-immunogenic mouse tumour models [11], nor from human cancer patients.

## 4. Pumps

Historically, the drug export transporter P-glycoprotein (P-gp) [12], encoded by the *ABCB1* (*MDR1*) gene in humans, has shaped ideas about MDR. This pump can remove a large range of drugs from the cell, and upregulation of P-gp makes it possible for cancer cells to become completely resistant to some of the drugs intensively used in the clinic, notably taxanes, anthracyclines, epipodophylotoxins and *Vinca* alkaloids [13]. Understandably, the discovery of a major form of MDR led to an optimistic sentiment in the field that all forms of drug resistance would soon be understood and, hence, overcome. From the start, it was clear, however, that even the versatile P-gp could only handle a limited number of amphipathic compounds that penetrate the membrane slow enough to be intercepted by an export pump. A host of other drugs—hydrophilic large drugs (methotrexate), nucleoside analogues (F-uracil) and nearly all alkylating agents—are poor P-gp substrates. The expectation that other pumps would turn up that would handle the drugs not transported by P-gp has also not materialized [14,15]. Some amphipathic drugs with low affinity for P-gp, such as the camptothecins/topotecan, are transported by BCRP (ABCG2) [16] and MDR Protein 4 (MRP4; ABCC4), but no general pumps have been found for alkylating agents [17]. Most of the transporters in the large ABCC (MRP) family have not been linked to resistance against anti-cancer drugs [18]. Where this is the case, the substrate specificity of these pumps overlaps with that of P-gp or BCRP.

Some 35 years after the first drug transporter associated with MDR was discovered, the sobering conclusion is that the evidence for a substantial role of these transporters in drug resistance in real tumours is limited. On the positive side, there is no doubt that modest upregulation of P-gp [19] or BCRP [20] can cause complete resistance to substrate drugs in a mouse model of human BRCA1-mutated breast cancer. Other transporters have not been found, however, as mediators of drug resistance in this model. The evidence for a role of any of these transporters in resistance of human cancers is largely negative as well. Effective inhibitors of P-gp have shown only limited effects in clinical trials [21,22]. There is no evidence that upregulation of other ABC transporters is consistently associated with drug resistance in human cancer patients. The lack of clinically useful inhibitors for BCRP or MRPs has precluded a more direct test of the possible contribution of those transporters to resistance.

Why these effective drug transporters are not more prominently used by human cancer cells in the defence against drugs can only be guessed. One reason could be that cancer patients are nearly always treated with drug cocktails that contain drugs not transported by ABC-transporters. Such tumours are primarily selected for resistance mechanisms that deal with all drugs simultaneously, rather than one of these drugs, and pumps will then not do. Another reason could be that the level of some of the most effective transporters is very low in many human tissues/tumours, lower than in mouse tissues/tumours. Hence, modest transcriptional upregulation of P-gp in human tissues does not result in transporter levels sufficient for resistance. Only drastic upregulation will help. Indeed, in the unusual cases where P-gp has been proved to contribute to resistance, the *ABCB1* (P-gp) gene in the tumour is hooked up to a strong promoter by a DNA rearrangement [23]. This is apparently a rare event. The *ABCB1* gene has not turned up as a gene predicting poor response to neoadjuvant chemotherapy of breast cancer [24,25]. Even the neo-adjuvant therapy, in which breast cancer patients are treated with anthracyclins or taxanes, has not resulted in substantial upregulation of P-gp [26] (J. de Ronde & L. Wessels 2012, personal communication), although this is the predominant mechanism of resistance against these drugs in a mouse model that closely resembles human breast cancer [27]. I think that these results show that not all drug resistance mechanisms are readily available in all tumours, not even powerful ones such as export pumps.

## 5. Residual disease: cell cycle effects

It has been known from the early days of experimental drug studies that cell cycle matters. This is hardly surprising. Most enzymes making DNA and RNA will stall at DNA damage. Non-cycling cells can take the time to repair the damage. DNA-damaging agents will primarily hit cells in the S-phase of the cell cycle and spindle poisons cells in mitosis. Hence, cells in the G0/G1 phase of the cell cycle have long been known to be relatively resistant to classical cytotoxic therapy [28]. Indeed, even cells sensitized to DNA damage by DNA repair defects are not uniformly killed by ionizing irradiation or alkylating agents: there is always a long tail in the dose–response curve [29]. Recent papers have explored the possibility that tumours may contain a fraction of quiescent cells that is actively kept in a (reversible) drug-tolerant state. I shall return to this a little later.

## 6. Residual disease: how do some leukaemia cells escape effective therapy?

Curative chemotherapy is seen only with some tumours with a high proliferative index—leukaemias, lymphomas, testicular cancer—and these are the exceptions to the rule that patients relapse, even if their tumours initially responded to chemotherapy. The most informative studies on the nature of the residual disease from which the relapse springs have been done with the inhibitors of signal transduction pathways, often called ‘targeted therapeutics’, a misnomer disregarding the exquisite targeting of some of the classical cytotoxic drugs, such as the topoisomerase poisons. In the case of kinase inhibitors, such as imatinib (Gleevec), a major mechanism of resistance is the presence of a small sub-population of leukaemic cells in which the ABL kinase, the target of imatinib, contains an amino acid substitution that prevents inhibition by imatinib [30]. Such target-altering mutations have also been observed with other kinase inhibitors [31]. The tumour may also avoid the deleterious effect of a road block in a signalling pathway to which it is addicted by activating an alternative pathway that circumvents the block. Such bypasses can explain resistance to HER2 or B-RAF inhibition.

This is not the whole story. If the only reason that CML caused by activation of ABL cannot be cured is the existence of minor fractions of tumour cells with an ABL kinase mutation, it should be possible to hit these sub-fractions with second- and third-generation ABL kinases inhibitors up front and to cure all CML [32]. If bypasses arise, around the drug-induced signal transduction block, it should be possible to inhibit these bypasses in turn with other drugs. Although successes have been scored by this approach [33], there are complications. One complication that seems to be especially relevant to leukaemias/lymphomas is the generation of blocks in cell death [34]. In a recent paper, massive upregulation of BCL6 was shown to protect an acute lymphoblastic leukaemia against cell death resulting from a block in ABL signalling. Inhibition of BCL6 sensitized the cells to ABL inhibitors [35]. A more general complication is the presence of a small fraction of cancer ‘stem’ cells, which is intrinsically resistant, because of its quiescence (see later text). If these cells are kicked into cycle by interferon-α, G-CSF or arsenic trioxide, they become sensitive to cytotoxic drugs [36–38]. Although this approach is theoretically appealing, its translation to the clinic has thus far given equivocal results [39] and factors other than quiescence may contribute to residual disease in leukaemia [40]. A slowly multiplying fraction of tumour cells was recently also identified in melanoma cells [41]. Expression of the H3K4 demethylase JARID1B is essential for the maintenance of this fraction, which displays tumour initiation ability. Although drug sensitivity of the slow-cycling melanoma cells has not yet been tested, the authors note that treatment with anti-cancer therapy *in vitro* results in the enrichment of the JARID1B-positive cells.

The study of residual disease is difficult in animals or humans. The cells are few, dispersed, and hard to isolate and characterize. This is why investigators have attempted to characterize a ‘residual disease’ fraction in cultured cells. In §7, I discuss prominent examples of this type of study.

## 7. A chromatin-mediated reversible drug-tolerant state in cancer cell sub-populations

Sharma *et al.* [42] treated a non-small-cell lung cancer (NSCLC) cell line with tyrosine kinase inhibitors (TKIs) targeting a mutant version of the epidermal growth factor receptor. Although the vast majority of the cells were killed, they obtained a small fraction of cells that survived drug concentrations 100-fold above the IC50. These ‘drug-tolerant persisters’ (DTPs) represent 0.3 to 5 per cent of the cell population and are not stably resistant. When grown in the absence of a drug, the cells rapidly regain drug sensitivity. A detailed investigation of the DTPs showed widespread alterations in gene expression, including several genes involved in chromatin modification, such as increased KDM5A/Jarid1A, a histone H3K demethylase and increased histone deacetylases (HDACs). Depletion of the KDM5A demethylase, or treatment of the cells with HDAC inhibitors, reduced the number of DTPs, indicating that the widespread chromatin modifications in the DTPs were responsible for resistance. The emergence of DTPs in these NSCLC cells required signalling via the IGF-1 receptor, as it could be prevented by an inhibitor of this receptor.

Sharma *et al.* [42] found DTPs in all tumour cell lines studied, including tumours originating from several different tissues. Although DTPs were initially isolated by their resistance to TKIs, DTPs are cross-resistant to cisplatin, suggesting generalized (pan-) resistance. Unfortunately, other drugs or X-rays were not investigated. The authors have also not yet determined how the DTPs arise or why these cells are resistant to drugs. One mode of generating slowly replicating cells was uncovered by Dey-Guha *et al.* [43]. In MCF7 cells multiplying *in vitro*, they observed occasional asymmetric divisions, in which AKT was downregulated in one of the daughter cells. This G0-like daughter then continued to replicate slowly. Inhibition of AKT led to increased formation of G0-like cells, and these cells were enriched in tumours after cytotoxic treatment of breast cancer patients, suggesting that the G0-like AKT-low cells could play a role in residual disease. Unfortunately, it is still unclear how (and why) cells decide to turn off AKT and generate a G0-like daughter. In another tumour cell line, upregulation of the ATF6α transcription factor promoted survival of dormant tumour cells in nude mice [44]. ATF6α is known to act as a survival factor after endoplasmic reticulum stress, and in this system it acts on mTOR via an AKT-independent pathway.

The notion that cancer can be associated with widespread epigenetic alterations is, of course, not new. Tumour suppressor genes can be turned off epigenetically [45], and attempts have been made to reverse this turn-off by DNA demethylating agents, by HDAC inhibitors and by combinations of both inhibitors, with modest clinical success [46]. There are also reports of synergistic effects of cytotoxic chemotherapy and HDAC inhibitors [47]. Hauswald *et al.* [47] have pointed out, however, that activation of silenced genes is a two-edged sword, as resistance genes may be activated as well. In AML cell lines, they showed that HDAC inhibitors activated the expression of a series of drug transporter genes resulting in a pleiotropic resistance phenotype extending far beyond classical MDR and including nucleoside analogues. ‘Epigenetic therapy’ will not be plain sailing.

Sharma *et al.* [42] have emphasized the resemblance of drug-tolerant cancer cells with ‘persisters’ in bacterial populations. I think that this resemblance is a spurious one, but I do not have space here to discuss this complex issue. Interested readers can find a justification for my scepticism in the electronic supplementary material, appendix SI.

When DTPs are kept under drug pressure, they eventually turn into ‘drug-tolerant expanded persisters’ (DTEPs), which can multiply in the presence of a drug. This is not residual disease but real resistance, and I shall return to the DTEPs below.

## 8. Are cancer stem cells the key to residual disease?

The cancer stem cell (CSC) concept has been succinctly summarized by Hans Clevers ([48], p. 313) in an elegant and critical review:

Central to the stem cell (CSC) concept is the observation that not all cells in tumours are equal. The CSC concept postulates that, similar to the growth of normal proliferative tissues such as bone marrow, skin or intestinal epithelium, the growth of tumours is fuelled by limited numbers of dedicated stem cells that are capable of self-renewal. The bulk of a tumour consists of rapidly proliferating cells as well as postmitotic, differentiated cells. As neither of these latter two classes of cells has the capacity to self-renew, the contribution of these non-CSC tumour cells to the long-term sustenance of the tumour is negligible.

It is not surprising that this CSC concept has fired the imagination of investigators working on drug resistance [49–51]. If tumours are driven by CSCs, the stem cells are the cells that need to be killed to eradicate the tumour. Incomplete eradication of cancer must leave some of the CSCs untouched and these are responsible for tumour relapse. Residual disease may therefore consist of stem cells equipped with specialized drug resistance mechanisms. It follows that chemotherapy aiming at cure should therefore target the CSCs rather than the bulk of the tumour cells [50,51]. To eliminate the weeds, you have to tear out the roots. If there were drugs that kill rare CSCs without touching the bulk of the tumour cells, they might even have been missed in standard chemotherapy trials.

Since the CSC concept was revived by Dick and colleagues for acute myeloid leukaemia in 1995 and extended to solid tumours in 2003, the concept has become the centre of heated controversies [52], as summarized by Clevers [48]. Some investigators think that the CSC concept should guide the search for new cancer therapies. In contrast, others believe that CSCs of solid human tumours are an artefact of the methods used to detect tumour-initiating cells (TICs). This requires dissociation of the tumour into single cells, fluorescence-activated cell sorting (FACS) and seeding in artificial niches in immunocompromised mice. This assay may select more for the ability of cells to survive extreme insults than for stemness. These sceptical investigators stress the flexibility of the tumour cell population, which allows more differentiated cells to dedifferentiate into CSCs. Obviously, if the CSC phenotype is not a stable trait, the development of drugs specifically targeting CSCs becomes less attractive [48,53]. If the phenotypic heterogeneity in tumours is reversible, as Morrison and co-workers have shown for melanomas [54], it becomes irrelevant to distinguish CSCs from the bulk population of cancer cells when considering targeted therapy [48].

Although the CSC concept has lost some of its lustre, it is still often invoked to explain residual disease. I shall therefore briefly summarize the evidence that CSCs have specialized defences against chemotherapy that could explain drug-resistant residual disease:

— *Drug transporters* [49]. It is often stated that stem cells, including CSCs, are rich in transporters able to extrude drugs from cells. This idea seems to have its origin in the haematopoietic stem cells, which indeed contain high concentrations of the two most versatile drug pumps, P-gp (MDR1, ABCB1) and BCRP (ABCG2). Initially, ABCG2 was even thought to be a general marker of stem cells, but more recent evidence has shown this to be incorrect. For instance, the normal mammary gland stem cell lacks ABCG2 [55,56]. Likewise, gut stem cells lack P-gp [57]. For other transporters present in CSCs, such as the MRPs (ABCCs), a generalized role in drug resistance is improbable. The MRP most generally present in cells, MRP1 (ABCC1), has never been conclusively linked to resistance in either mouse model tumours or human clinical samples [49]. Even if high levels of a MDR-type drug transporter are found in some CSCs, these can only explain resistance to substrates of the transporter, not to the many prominent drugs not touched by MDR transporters, as also pointed out by Dean [49].
— *Resistance to DNA damage*. The most unambiguous results have been obtained with ionizing radiation, which is not complicated by target alterations (e.g. topoisomerase down regulation) or drug uptake problems encountered by DNA-interacting drugs. The CD133-expressing glioma cells with CSC properties are more resistant to ionizing radiation than the CD133-negative tumour cells [58] and the same holds for the putative CSC fraction of human breast cancer [59]. Why is not known. It could be due to more efficient repair of DNA strand breaks, or to more CSCs being quiescent-like, allowing more time for DNA repair before cells enter S-phase and find their DNA too damaged to survive DNA replication.
— *Quiescence*. A low rate of multiplication is a hallmark of the somatic stem cells of normal tissues, the majority of colon epithelial stem cells being the exception [48,60]. Whether this makes stem cells less vulnerable to chemotherapy is not self-evident. Blanplain and co-workers [61] have claimed that being in G0/G1 when your DNA gets hit can actually be unhealthy for a stem cell, as duplex breaks in DNA cannot be repaired by the error-free homology-directed system only available during and after DNA replication. Instead, the error-prone system of non-homologous end joining has to be used to fix duplex DNA breaks. Nevertheless, the generally accepted hypothesis is that quiescence of stem cells protects against cytotoxic therapy [48,62–64]. The presence of quiescent cells with CSC properties has been demonstrated in several tumour systems, using retention of DNA label or lipophilic dye. Whether these are the cells in the tumour that result in residual disease and whether their quiescence is responsible for their survival remains to be seen. The most convincing experiments have been published by Andreas Trumpp and co-workers [36,37], who showed that leukaemia stem cells could be targeted by breaking their dormancy.
— *Epithelial to mesenchymal transition* (*EMT*). There is no doubt that EMT provides a formidable version of pan-resistance [65] and I shall return to this below. The question here is whether residual disease is due to EMT. This question is not easily answered, as EMT can be a transient state that could be easily missed. Moreover, residual disease is usually poorly accessible to detailed analysis, and often the analysis does not include an evaluation of EMT. In the few model systems in which this was verified, no EMT was found [66] and EMT therefore does not appear to provide a general explanation for residual disease.

Are CSCs responsible for the therapy-resistant fraction resulting in residual disease? This is obviously the key question. There are now several tumour systems in which CSC-like cells are enriched in tumour remnants after therapy. These include gliomas, breast cancer, colon cancer and a sophisticated CML mouse model [48]. In our laboratory, Pajic *et al.* [66] have studied the issue in a conditional mouse model of human triple-negative breast cancer. In the mouse, the somatic stem cells are well defined in normal mammary glands. Cells with the same surface markers proved to be highly enriched in the tumour-initiating fraction isolated from the tumour. However, the few cells in this tumour repeatedly surviving cisplatin therapy were not enriched in these TICs. This raises the question whether residual disease in other tumour systems is really due to putative CSCs or a consequence of other properties of CSCs, such as quiescence, allowing them to survive drug treatment.

A major effort is under way to find drugs that preferentially target stem cells [67]. As pointed out by Clevers [48], the initial ideas driving this effort were too simple. Tumours have no roots that one can specifically tear out, dooming the plant. There is little doubt that some of the more differentiated tumour cells can dedifferentiate to replace the killed CSCs. If CSC-targeted therapy is going to make a contribution, it is only in conjunction with therapy targeting the bulk of the tumour.

Zhou *et al.* [51] and Frank *et al.* [68] have written detailed and optimistic reviews of the new therapeutic opportunities provided by the CSC hypothesis. The drugs under development mainly attempt to target signalling pathways involved in the regulation of self-renewal of normal somatic stem cells, such as the Wnt, the Sonic Hedgehog and the Notch pathways. The drugs should either preferentially block stem cell (and CSC) renewal or drive the stem cells into differentiation, closing down the tumour supply line. As the authors point out, a major problem is specificity, as with all tumour chemotherapy. Indeed, the only small molecule that targets a pathway involving stem cell self-renewal and that has managed to reach a phase II trial at the time the review of Zhou *et al.* [51] was written is a SMO (Sonic Hedgehog) antagonist. This was developed, however, for patients with basal cell carcinoma, most of whom have mutations in Hedgehog pathway components [69].

Other approaches attempt to target surface molecules preferentially present on CSCs [68]. Whether the (limited) effectiveness of these antibodies against metastatic cancer is due to their targeting of CSCs remains to be seen.

## 9. Residual disease: conclusions

In summary, of the many different explanations advanced for residual disease: the old-fashioned one seems best supported by experimental data: residual disease is due to quiescent cells. These cells are not cells that just happen to be in G1, but cells that have entered a specific quiescence programme. This programme may involve widespread alterations in gene expression that are reversible, allowing these cells to re-enter the cell cycle when danger is gone. The analogy of these quiescent cells with bacterial ‘persisters’ is misleading, in my opinion, as explained in the electronic supplementary material. Residual disease in cancer is not the expression of a genetic programme that protects the population from total destruction. It is a state of a small fraction of the tumour cells that allow these cells to avoid being killed.

## 10. Pan-resistance: general considerations

The most frustrating and intractable form of resistance is pan-resistance; resistance to any drug, and often also to ionizing radiation. It is as if the cancer cell has lost all targetable defects. Some targets can indeed be lost, as carcinogenesis can be a hit-and-run process. For instance, DNA repair defects are mutagenic and contribute to tumourigenesis, but full-blown tumours do not need the defect to continue growing. Hence, deficiencies in homology-dependent DNA repair caused by downregulation or mutation of *BRCA1/2* can be reversed in the mature tumour during drug treatment. Methylation of the promoter may be reversed; chain-terminating mutations can be mitigated by a second mutation restoring the reading frame [70]. Although this removes an obvious Achilles heel of the cancer cell that made it vulnerable to drugs, the basic hallmarks of the cancer cell are not altered and it continues to proliferate. Why are there no obvious activated growth promoting pathways or failed cell cycle checkpoints in pan-resistant cells that can be exploited by available drugs? Quiescence is not an explanation: the tumour continues to proliferate, whatever the medical oncologist throws at it.

Basically, there are three types of explanations for pan-resistance: mimicry, superior defence or compensation. Mimicry of normal cells entails the adoption of proliferation strategies of normal cells. Rather than relying on abnormal activation of proliferation-promoting pathways, the cancer cell blends in by imitating rapid growth of host tissues with high turnover rates, making the tumour equally sensitive/resistant to drugs as normal tissues. This is a theoretical possibility that I find implausible. Normal cells are completely dependent on external signals for growth and it seems unlikely that a tumour would be able to exploit normal exogenous growth stimuli without the cost of any targetable vulnerability. Nevertheless, I mention this possibility to re-emphasize that tumour cells responding to drug are hypersensitive to that drug relative to the normal cells in the body. If they lose that hypersensitivity and become ‘resistant’ to all drugs, they have just levelled the playing field. And on a level field, the cancer cell wins.

The two other explanations for pan-resistance—superdefence and compensation—may seem two sides of the same coin, but they are not. Superdefence is the ability to keep all drugs away from their targets. P-gp upregulation could be part of such a superdefence system. P-gp does not affect drug targets in the cell or the vulnerability of the tumour cell to drugs hitting those targets. P-gp only prevents the drug from reaching its target. In contrast, compensation represents adaptations that affect multiple targets in an indirect way without influencing drug–target interaction (i.e. increased DNA repair, or less dependence on a growth-promoting activated signal-transduction pathway by activation of a parallel pathway). The optimistic view is that superdefence or compensatory adaptations both involve alterations in gene expression that might be exploited by drug treatment. Upregulated pumps can be targeted with inhibitors; there are even attempts to develop drugs that specifically hit cells with upregulated P-gp [71]. Activated parallel pathways may be targeted with additional drugs. Once we know how the cancer cells avoid destruction by therapy, it is hoped that we can adapt our therapeutic strategy.

Pan-resistance is tough to study. The most useful information comes from model systems in which initial sensitivity to drugs is replaced during treatment by pan-resistance. Unfortunately, pan-resistance is usually accompanied by massive alterations in gene expression, making it hard to pinpoint which changes are actually responsible for the resistance. I will discuss here the most informative systems studied.

## 11. Chromatin-mediated pan-resistance

Sharma *et al.* [42] have found a chromatin-mediated reversible drug-tolerant state in cancer cell lines grown *in vitro*, as discussed earlier. Although these ‘DTPs’ are largely quiescent, approximately 20 per cent of DTPs eventually resume normal proliferation in the presence of a drug, yielding ‘DTEPs’, which can be propagated in drugs forever [42]. DTEPs, like DTPs, can be obtained from very different cell lines and display widespread alterations in gene expression. The drug-tolerant state of DTEPs is also unstable, but reversion to drug sensitivity takes about 90 cell doublings, showing that the drug-tolerant state has become stabilized to some extent in the DTEPs. Like DTPs, formation of DTEPs can be inhibited by downregulation of the histone H3K4 demethylase KDM5A and by inhibition of HDAC activity. Cells originally selected for resistance to TK inhibitors proved cross-resistant to cisplatin, indicating a broad mechanism of resistance, although no other drugs were apparently tested. Why DTEPs are resistant is not known. They are not really pan-resistant, as treatment with HDAC inhibitors induces a DNA-damage response that kills the cells. It should also be noted that resistance in this system has only been studied *in vitro*. Whether the resistance observed in test tubes is sufficient to make the tumour resistant in animals remains to be seen. Nevertheless, this is an extremely interesting and tractable system to study mechanisms of drug resistance that are not simply caused by target loss or pump upregulation.

## 12. Blocks in apoptosis or necroptosis

For a time, blocks in apoptosis were popular as an explanation for drug pan-resistance. The concept is simple: drugs may kill cancer cells by activating programmed cell death. Cells that would inactivate that programme would obviously be more resistant to killing and buy time for damage repair. There was also appealing evidence to support the theory. Scott Lowe and his collaborators [72] used a mouse lymphoma model, driven by an activated *Myc* gene, that responded to cytotoxic drugs with apoptosis. Inactivating the apoptotic programme resulted in resistance. The versatile combination of *in vitro* and *in vivo* experiments possible in this lymphoma system resulted in a series of landmark papers that established that blocks in apoptosis could reduce drug-induced cell kill, at least in a lymphoma model highly susceptible to apoptotic death. In this model, blocks in apoptosis also diminish susceptibility to alkylating agents or X-rays. The resistance is therefore a true pan-resistance.

It is in the generalization of this appealing concept to tumours of epithelial origin in human patients that problems arose. These problems have been discussed in detail [72,73] and will not be reiterated here. The essence was summarized by Brown & Attardi ([74], p. 236):

To become malignant, the cell must inactivate the apoptotic pathway. As a consequence, the cell's susceptibility to apoptosis is severely compromised and other forms of death become more important for cell killing and tumour response to DNA-damaging agents.

This point has also been stressed by Blagosklonny [75].

Interestingly, even in the apoptosis-prone lymphoma model of Lowe and co-workers [76], more recent experiments have tended to de-emphasize blocks in apoptosis as a cause of drug resistance. Resistance to doxorubicin was found to be caused by downregulation of *p53*, *Chk2* and *Top2*α** (encoding topoisomerase II, the target of doxorubicin); resistance to camptothecin was caused by downregulation of *Top1* (encoding topoisomerase 1, the target of camptothecin). No evidence was found either for blocks in apoptosis as a cause of resistance to a range of drugs in a conditional breast cancer model in mice [77]. Attempts to improve cancer chemotherapy in human cancer patients by inhibiting apoptosis have not resulted in new standard treatments.

Since the early attempts to target apoptosis resistance in cancer, a second programmed cell death pathway has been characterized: necroptosis [78,79]. This involves leakage of lysosomes and an explosive rupture of the dying cells. Necroptosis is a key process in chronic inflammatory diseases, but its role in cancer remains to be defined. Lethally damaged cancer cells die by mitotic catastrophe or necrosis, if not by apoptosis, but whether this necrosis is programmed and involves the necroptosis pathway remains to be seen.

It is possible that cancer usually entails an effective inactivation of all programmed death pathways and that this inactivation is not complete in the exceptional tumours that are cured by chemotherapy, such as leukaemias, lymphomas or testicular cancer, as suggested by Blagosklonny [75]. Also, tumours that are not cured often shrink under chemotherapy and the cells appear to die by necrosis. It remains possible that this process can be promoted by chemotherapy. Cell kill by drugs or X-rays is often increased by inhibition of signal transduction pathways [62], possibly interfering with the ability of pro-survival signalling to promote DNA repair [62]. The possibility that tumour kill could be enhanced by reducing blocks in programmed death pathways remains an interesting one and retains ardent proponents [80].

## 13. Epithelial to mesenchymal transition

The EMT in carcinomas is invariably associated with the resistance to a variety of anti-cancer drugs. Notwithstanding a major worldwide effort to dissect mechanisms of drug resistance associated with EMT, the picture is still foggy. The reason is that EMT results in a massive reprogramming of gene expression and it is difficult to sort out which alteration is essential for each form of resistance [65]. Although it is easy to demonstrate that EMT is associated with upregulation of drug export pumps or of DNA repair mechanisms in cultured cell lines [81], the relevance of these mechanisms for the high degree of resistance of EMT tumours *in vivo* is not known. EMT results in cells with properties resembling CSCs [65]. Hence, all drug resistance mechanisms invoked for stem cells are also proposed for cancer cells that have undergone EMT [82]. This is not very helpful, as it is not clear why CSCs are drug-resistant or even whether this is always the case in real tumours, as mentioned previously. There are new experimental tumour models resembling human tumours that are accessible to detailed molecular studies. For instance, Rottenberg & Jonkers (2012, personal communication) have found that about half of the mammary carcinomas arising in the p53, BRCA2-deficient mouse model [83] undergo EMT and become unresponsive to chemotherapy. Although this is a powerful system to study EMT-associated drug resistance, these studies will not be plain sailing. Finding the alterations responsible for resistance remains a search for needles in the vast haystack of gene expression alterations.

## 14. Outlook

A vast amount of information is being published on drug resistance mechanisms and on methods to restore sensitivity to resistant cells using isolated cell lines. In the more ambitious papers, attempts are made to couple this information to the response of tumours in patient samples or in experimental mouse tumours. Often, however, only cell lines are studied. It is nearly always easier to kill tumour cells in a test tube than in real tumours. Often there is doubt whether results obtained in established cell lines can be extrapolated to the behaviour of these cells when they were still in a tumour [84]. This is why clever attempts to increase the effectiveness of chemotherapy often fail clinically. From my summary of the literature it should be clear that we still do not know why. The most reasonable interpretation of the cause of drug-resistant residual disease is the presence of (semi)-quiescent cells; for pan-resistance there is not even a generally accepted plausible hypothesis. I agree, however, with Ira Mellman, vice-president for oncology research of Genentech, that epigenetics is ‘the sleeping giant of drug resistance’ (2012, personal communication). The field of drug resistance has always been dominated by mutations in analogy to bacterial resistance [85,86]. Mutations cause resistance; selection of mutants results in the emergence of resistance. The demonstration that cells can become pan-resistant owing to widespread epigenetic alterations and that such alterations can occur at a much higher frequency than mutations is a major breakthrough, even though the mechanism of resistance is still unclear.

Although studies on cell lines remain essential for adding to our knowledge of drug resistance mechanisms [87], it seems obvious that the ins and outs of residual disease and pan-resistance can only be solved in tractable animal models, which resemble human cancer sufficiently to allow extrapolation of the results obtained to human disease. The mammalian genome is finite and the number of drug resistance mechanisms is finite as well. Given the power of DNA and RNA sequencing, proteomics and bioinformatics, the job will get done. Genetically modified mouse models should provide the answers to even the most difficult questions [9,83,88–90].

## Supplementary Material

Persisting drug-tolerant cells
