# Supplementary material for: Cancer drug pan-resistance: pumps, cancer stem cells, quiescence, epithelial to mesenchymal transition, blocked cell death pathways, persisters or what?
Source: Open Biol. 2012 May;2(5):120066. doi: 10.1098/rsob.120066 (PMC3376736; doi:10.1098/rsob.120066)
Supplement: Persisting drug-tolerant cells [file rsob120066-s1.doc]

# Supplementary Material

# Persisting, drug-tolerant cells. A comparison of bacteria and cancer cells.

by Piet Borst

## Introduction

The phenomenon of drug-sensitive cells that are nevertheless not killed by drug has long been known in bacteria. Such cells are called persisters and their ability to survive lethal drug concentrations is called tolerance. If the persistent cells are allowed to grow out, they are killed but for a small fraction of persisters. Sharma et al. (1) have recently pointed at similarities between drug-tolerant cancer cells and persisters in bacterial populations and this similarity has been embraced in several recent review papers (2) (3) (4). Although I agree that we can learn as cancer biologists from bacterial drug resistance, I think that the analogy breaks down when one looks at the details. Here I summarize what is known about bacterial persisters and whether they resemble persisting cancer cells. A substantial fraction of my text has simply been copied from 2 excellent reviews (5) (6).

## A summary of bacterial persistence

The introduction of a recent paper (5) gives a concise summary: “persister cells are multidrug-tolerant cells that contribute to the antibiotic recalcitrance of biofilm infections. They are survivor cells that make up a small part of the population: 10^-6^ to 10^-4^ in exponentially growing cultures and ~10^-2^ in stationary phase. Persisters are tolerant to all currently available antibiotics and are found in all species examined. Persisters are nongrowing, dormant cells; persistence is not heritable; persisters probably form a single subpopulation tolerant to unrelated antibiotics; a transcriptome of isolated persisters points to toxin/antitoxin modules as possible persister genes; and expression cloning points to enzymes of phospholipid synthesis being involved in persister maintenance.”
 In general, non-growing or slow-growing bacteria are less sensitive to antibiotics. This property is called ‘drug indifference’. Indifference is not the same as persistence. It is a reflection of the overall reduced sensitivity of dormant/slow-growing microbial populations, with no specific mechanistic basis. In contrast, persistence is a property of a small sub-fraction of the culture, whether growing or not.

***Genetics of bacterial persistence (6;7)*** The study of persistence has been hampered by 2 complications: (1) Persisters are only a small fraction of the population, making it tough to collect pure persister cells for analysis. (2) There are several ways in which cells can enter the persister state. This has complicated the isolation of mutants with strong phenotypes, be it increased or decreased formation of persisters.
 The first useful mutant was a high persister (*hip*) mutant of *E. coli* isolated by Moyed and co-workers. The *hip* locus consists of *hipA* and *hipB*. The best studied allele of *hipA*, *hipA7*, raises the % of persisters 10,000 fold to 1% of the population. The *hipA7* mutant displays increased persistence to all kinds of damage, including DNA damaging agents and heat.
 The *hip* operon is a TA module, *i.e.* a toxin-antitoxin genetic element. There are several of these elements in *E. coli*. They appear to be non-essential, like the *hip* operon, and most investigators think that these elements are specifically involved in generating persistence. *HipA* encodes the toxin and this is attributed to its kinase activity. Initially, HipA was thought to be a member of the PI-3/4 kinase family, but more recently it was found to be a protein kinase that can autophosphorylate. One of the cellular targets of HipA appears to be EF-Tu and Schumacher et al. (8) present some evidence indicating that HipA-mediated EF-Tu phosphorylation blocks EF-Tu function (*i.e.* catalyzing aa-tRNA binding to the ribosome). This would inhibit protein synthesis, explaining dormancy. However, since the gene expression profile of the *hipA7* persister mutant shows 300 overexpressed genes as well as many downregulated genes, it is doubtful whether EF-Tu is the only target of HipA. The requisite mutagenesis experiments on EF-Tu to remove the phosphorylated target amino acid of HipA have not been done.
 HipB is both an inhibitor of HipA, forming a tight HipA-HipB complex and a repressor of the Hip operon. The HipA7 mutant contains a single aa substitution in HipA that weakens its interaction with HipB, explaining the increased level of persisters in the mutant population (8).
 A generalized hunt for genes affecting persistence used either moderate overexpression of a genomic library, or a genome-wide transposon gene disruption (5). This yielded what Lewis (6) calls a “plethora of multidrug tolerance” genes. Most effector genes involved in persistence stop gene expression at the level of protein synthesis or RNA stability. This is illustrated by a recent paper (9), which shows that the ubiquitous Lon (Long Form Filament) protease and mRNA endonucleases (mRNases) encoded by toxin-antitoxin (TA) loci are required for persistence in *E. coli*. Successive deletion of the 10 mRNase-encoding TA loci of *E. coli* progressively reduced the level of persisters, showing that persistence is a phenotype common to TA loci. Maisonneuve et al (9) propose that the mRNAses encoded by the TA loci are activated in a small fraction of growing cells by Lon-mediated degradation of anti-toxins. Degradation of mRNA stops translation and induces a reversible state of dormancy and persistence.

The complex set of toxins that can stop bacterial protein synthesis in which these mRNAses participate, was presumably selected in evolution to kill competitors/predators. In persistence this program is used to temporarily shut down endogenous protein synthesis to allow the bacterial population to escape from lethal environmental insults, such as antibiotics.

***Regulation of persistence***

“Examination of the rate of *E. coli* persister-cell formation over time showed that few of these cells are formed in early exponential phase, followed by a sharp increase in persister-cell formation in mid-exponential phase, reaching a maximum of ~1% of cells forming persisters in the non-growing stationary phase. In order to probe whether persisters isolated during the early exponential phase of growth were introduced from a stationary state culture inoculum, or were formed *de novo*, the culture was kept in early exponential phase by repeated dilution and regrowth. After four cycles, persister cells completely disappeared. This simple experiment rules out non-specific mechanisms of persister formation and indicates that persister cells are preformed, rather than being produced in response to antibiotics” (6).

“Persisters comprise a small sub-population, and in mid-exponential phase *E. coli* produces as few as 10^-5^ surviving antibiotic-tolerant persister cells. Given that all of the cells in a population are genetically identical it seems that persisters must be produced by a stochastic process. Indeed, what is the alternative? Fluctuations in the concentrations of a small number of dedicated proteins are probably responsible for the formation of persisters. The absence of persisters in an early exponential population might be due to low concentrations of persister proteins at this stage. It seems that two processes control persister formation – a stochastic fluctuation in the level of specific proteins (persister proteins), and a controlled, regulated mean level of expression of these proteins, which is dependent on the density of the population, and probably on several other factors as well. The environment therefore sets the baseline over which stochastic fluctuation of expression will take place” (6).
 “As persisters do not grow, it would seem logical for the entire stationary population to enter into this protected survival state, but this is not the case. The benefit of being a regular, dividing cell seems to stem from its ability to rapidly resume growth, a process that takes 1 – 1.5 hours longer for a dormant persister cell. Because most cells in an *E. coli* or *P. auruginosa* stationary phase population are non-persisters, this indicates that the optimal individual strategy is not to enter into persistence, suggesting that the persister state is an altruistic behaviour benefiting the kin. Indeed, in the rare instance that the whole population of normal cells is killed by a lethal factor, the surviving persisters can propagate the genome that they share with their kin.” (6)
 Persistence can be considered “as an insurance policy to guarantee population survival when faced with catastrophic situations, such as antibiotic exposure” (7). Persistence genes are therefore contingency genes (10) that “facilitate the efficient exploration of phenotypic solutions to unpredictable aspects of the host environment” (10), and it is surprising that scientists working on persistence have not made the link to contingency. Known contingency genes encode surface antigens and resistance genes and they can be switched by low-frequency stochastic events. These events often involve DNA rearrangements, but epigenetic mechanisms, such as the transcriptional activation of the var genes in *Plasmodium*, also occur (reviewed in ref. (11)).
 When contingency genes are controlled by recombinases, such as inversion of DNA segments, the stochastic nature of the event appears to be controlled by fluctuations in recombinase (and accessory protein) levels and by the statistics of rare associations between recombinase enzyme and DNA target (11). What sets the level of persister generation will be difficult to determine, given the many roads that appear to lead to the Rome of persistence.

***Persistence of bacterial and cancer cells compared*** Obviously, there is a fundamental difference between populations of bacteria and populations of cancer cells. Bacteria have been selected at the population level for survival. Hence, the heavy investment in contingency persistence genes. Although detrimental under optimal conditions, the minor fraction of persisters/switchers saves the population from total annihilation when catastrophe strikes. The (stochastic) pre-adaptation is costly, but necessary. Inducible defense is cheaper, but will often come too late. When a lethal antibody against a bacterial surface component is made by the mammalian host, it is too late for the bacteria to start the (time-consuming) process of surface coat replacement. The same often holds for antibiotics or DNA damage.
 In contrast, tumor cells are egotistic. They have escaped from the yoke of tissue control and go it alone. A tumor is a population of competing cells involved in a Darwinian struggle, each cell fighting for itself. Although it would be of great benefit to the population to evolve contingency measures, I do not see how this could ever occur in practice. Tolerance and persistence of tumor cells therefore reflect the properties of individual cells, the ability of the cell to avoid death under exceptional circumstances using mechanisms to shut down growth and avoid excessive lethal damage responses that are in principle available in all normal cells, but overruled in these cells by dominant mechanisms that eliminate severely damaged cells.

The analogy between persisting bacterial and persisting cancer cells is therefore that both are able to lie low and avoid the processes that allow lethal damage to take its toll.

However, the analogy breaks down when one looks at the details. Persistence in bacteria is a highly selected survival mechanism operating at the population level and involving specific contingency genes controlled by stochastic mechanisms. Persistence in cancer cells is a trick of individual cells to survive damage. The nature of this trick is not known. It could be accidental lack of growth, similar to the drug indifference of bacteria in stationary phase. It could be a specialized (induced) damage response, only successful in part of the tumor cell population. It could be a specific cellular state that infrequently occurs and that allows the cell to tolerate lethal damage without activating a death response.

Undoubtedly, we can learn in the cancer field from bacterial resistance mechanisms, as pointed out by Sharma et al. (1) and other investigators (2) (3) (4). Bacterial persistence shows the enormous diversity and complexity of epigenetically controlled resistance mechanisms, even in simple organisms as bacteria. As argued here, however, to equate bacterial persistence with drug-tolerant cancer cells goes one step too far.

Reference List

(1) Sharma SV, Lee DY, Li B, Quinlan MP, Takahashi F, Maheswaran S, et al. A chromatin-mediated reversible drug-tolerant state in cancer cell subpopulations. Cell 2010 Apr 2;141(1):69-80.

(2) Dawson CC, Intapa C, Jabra-Rizk MA. "Persisters": survival at the cellular level. PLoS Pathog 2011 Jul;7(7):e1002121.

(3) Glickman MS, Sawyers CL. Converting cancer therapies into cures: lessons from infectious diseases. Cell 2012 Mar 16;148(6):1089-98.

(4) Lambert G, Estevez-Salmeron L, Oh S, Liao D, Emerson BM, Tlsty TD, et al. An analogy between the evolution of drug resistance in bacterial communities and malignant tissues. Nat Rev Cancer 2011 May;11(5):375-82.

(5) Hansen S, Lewis K, Vulic M. Role of global regulators and nucleotide metabolism in antibiotic tolerance in Escherichia coli. Antimicrob Agents Chemother 2008 Aug;52(8):2718-26.

(6) Lewis K. Persister cells, dormancy and infectious disease. Nat Rev Microbiol 2007 Jan;5(1):48-56.

(7) Jayaraman R. Bacterial persistence: some new insights into an old phenomenon. J Biosci 2008 Dec;33(5):795-805.

(8) Schumacher MA, Piro KM, Xu W, Hansen S, Lewis K, Brennan RG. Molecular mechanisms of HipA-mediated multidrug tolerance and its neutralization by HipB. Science 2009 Jan 16;323(5912):396-401.

(9) Maisonneuve E, Shakespeare LJ, Jorgensen MG, Gerdes K. Bacterial persistence by RNA endonucleases. Proc Natl Acad Sci U S A 2011 Aug 9;108(32):13206-11.

(10) Moxon ER, Rainey PB, Nowak MA, Lenski RE. Adaptive evolution of highly mutable loci in pathogenic bacteria. Curr Biol 1994;4:24-33.

(11) Borst P. Mechanisms of antigenic variation: an overview. In: Craig A, Scherf A, editors. Antigenic Variation.London: Elsevier Ltd; 2003. p. 1-15.
